# High-Accuracy Maize Disease Detection Based on Attention Generative Adversarial Network and Few-Shot Learning

**DOI:** 10.3390/plants12173105

**Published:** 2023-08-29

**Authors:** Yihong Song, Haoyan Zhang, Jiaqi Li, Ran Ye, Xincan Zhou, Bowen Dong, Dongchen Fan, Lin Li

**Affiliations:** 1 China Agricultural University, Beijing 100083, China; 2020319010229@cau.edu.cn (Y.S.); zhanghaoyan0526@163.com (H.Z.); lijiaqi@cau.edu.cn (J.L.); yeran@cau.edu.cn (R.Y.); zxc0119@cau.edu.cn (X.Z.); dongbw@cau.edu.cn (B.D.); 2 School of Computer Science and Engineering, Beihang University, Beijing 100191, China; 213352411@buaa.edu.cn

**Keywords:** maize disease detection, attention mechanism, generative adversarial network, few-shot learning

## Abstract

This study addresses the problem of maize disease detection in agricultural production, proposing a high-accuracy detection method based on Attention Generative Adversarial Network (Attention-GAN) and few-shot learning. The method introduces an attention mechanism, enabling the model to focus more on the significant parts of the image, thereby enhancing model performance. Concurrently, data augmentation is performed through Generative Adversarial Network (GAN) to generate more training samples, overcoming the difficulties of few-shot learning. Experimental results demonstrate that this method surpasses other baseline models in accuracy, recall, and mean average precision (mAP), achieving 0.97, 0.92, and 0.95, respectively. These results validate the high accuracy and stability of the method in handling maize disease detection tasks. This research provides a new approach to solving the problem of few samples in practical applications and offers valuable references for subsequent research, contributing to the advancement of agricultural informatization and intelligence.

## 1. Introduction

With the growth of the global population, the importance of food production is becoming increasingly prominent. As one of the most widely cultivated and highest yielding food crops in the world, maize plays a significant role in the global food supply [1]. However, problems due to disease in maize production have always been one of the major challenges faced in agricultural production. Maize diseases not only directly affect the yield of maize but also reduce the quality of maize, further affecting farmers’ income and global food security [2]. Traditional maize disease detection methods mainly rely on manual observation and identification, which are not only inefficient but also limited by manual experience and skills [1]. In recent years, with the development of computer vision and machine learning technologies, using these technologies for disease detection has become a new trend [2,3,4,5]. However, these methods usually require a large amount of labeled data to train the model, and it is often difficult to obtain a large amount of labeled data in practical applications, especially for specific tasks such as agricultural disease detection.

In the realm of crop research grounded on traditional machine learning techniques, an array of studies has been carried out. For instance, traditional machine learning methods such as Linear Regression (LR), Random Forest (RF), and Support Vector Machine (SVM) were utilized by Jody Yu et al. [6] and Hwang Lee et al. [7] to assess multispectral imagery, enabling the estimation of nitrogen content in maize canopies. Moreover, a demonstration of the predictive performance for large-scale maize height mapping, with an RMSE approximately between 40 and 50 cm, was presented by Qinghua Xie et al. [8], leveraging crop height retrieval technology based on radar data. Pound et al. [9] extracted combined features from images, subsequently employing classifiers such as Linear Discriminant Analysis and SVM for classification training. Furthermore, the deployment of Convolutional Neural Network (CNN) was seen in the work of Balaji et al. [10], with the optimization of its parameters aiding the study of plant disease monitoring, thereby significantly improving detection speed and accuracy. It is to be noted that all these studies relied on datasets of substantial volume. In this context, few-shot learning provides a new solution. Few-shot learning is a machine learning method aimed at understanding and recognizing new categories by learning a small number of samples [11]. This method has a wide range of applications in many fields, such as image recognition [12], natural language processing, etc. [13]. In agricultural disease detection, few-shot learning can effectively solve the problem of data scarcity and improve the efficiency and accuracy of disease detection.

Therefore, this paper will introduce a high-precision maize disease detection method based on Attention Generative Adversarial Network (Attention-GAN) and few-shot learning. The proposed method introduces the attention mechanism, enabling the model to better focus on the key parts of the image, improving the accuracy of disease detection. At the same time, the Generative Adversarial Network (GAN) [14] is utilized to enhance the training data. These processes further improve the generalization ability of the model. Finally, the few-shot learning method is employed, allowing the model to effectively detect diseases using only a small amount of labeled data.

This research is not only of great significance for improving the accuracy of maize disease detection but also has important value for promoting the development of agricultural intelligence, improving agricultural production efficiency, and ensuring global food security. It is hoped that through this research, a new and effective solution for agricultural disease detection can be provided, offering strong technical support for agricultural production practice.

## 2. Related Work

### 2.1. Attention Mechanism

The attention mechanism is a crucial technique in machine learning, where the central idea is to focus more on the parts of the data that are more relevant to the current task when processing data [15,16]. This mechanism was first widely used in the field of natural language processing, especially in machine translation tasks, where it can help the model focus more on the relevant parts of the source language sentence when translating a word or phrase [17]. In the field of computer vision, the attention mechanism is also widely used in various tasks such as image classification, object detection, semantic segmentation, etc. In these tasks, the attention mechanism can help the model better understand the content of the image, extract more effective features, and thus improve the model’s performance.

In this paper, the attention mechanism is applied to maize disease detection. Identifying the diseased parts from the images of maize is a very complex task because the diseased parts may only occupy a small part of the image, and the shape, color, texture, and other features of the disease may greatly differ from those of healthy parts. Therefore, a model that can focus attention on the diseased parts is needed, and the attention mechanism can meet this requirement. The basic form of the attention mechanism can be represented as a weight distribution function, which determines the parts that the model should pay attention to when processing the input data [15]. Specifically, for an input sequence $X=\{x_{1},x_{2},...,x_{n}\}$, the attention mechanism first calculates an attention score $e_{i}$, then converts these scores into weights $a_{i}$ through a softmax function, and finally multiplies each element of the input sequence by its corresponding weight to get the weighted sequence $Z=\{z_{1},z_{2},...,z_{n}\}$. This process can be represented by the following mathematical formulas:

(1) $$e_{i}=f\left(x_{i}\right)$$

(2) $$a_{i}=\frac{exp(e_{i})}{\sum_{j=1}^{n}exp\left(e_{j}\right)}$$

(3) $$z_{i}=a_{i}*x_{i}$$

where f(·) is a learnable function used to calculate the attention score. In practical applications, f(·) can be a fully connected network, convolutional network, recurrent network, etc.

### 2.2. Generative Adversarial Network

Generative Adversarial Network (GAN) is a deep learning model proposed by Ian Goodfellow et al. in 2014 [14]. The core idea of GAN is to train generative models in an adversarial manner, enabling them to generate fake data similar to real data. The basic structure of GAN includes two parts: the generator and the discriminator. The task of the generator is to generate fake data, and the task of the discriminator is to judge whether a piece of data is real or fake. During the training process, the generator and the discriminator learn in an adversarial manner. The generator tries to generate increasingly realistic fake data to deceive the discriminator, while the discriminator tries to identify fake data more accurately. This process can be vividly described as a two-person zero-sum game, where the generator and the discriminator are like two adversarial players, improving their abilities in the game. In the field of computer vision, GAN is widely used in various tasks such as image generation, image super-resolution, image style transfer, etc. In these tasks, GAN can generate high-quality images, and even generate fake images that are almost indistinguishable from real images.

In the task of this paper, GAN is applied to maize disease detection. GAN is used to generate images of maize diseases, and these images are used to train the disease detection model. This can greatly increase the amount of training data and improve the performance of the model. In addition, various types and degrees of disease images can be generated using GAN, enabling the model to handle more complex and diverse disease situations. The training process of GAN can be represented by the following mathematical formula:

(4) $$\min_{G}\max_{D}V\left(D,G\right)=E_{x∼p_{\mathrm{data}}\left(x\right)}\left[\log D\left(x\right)\right]+E_{z∼p_{z}\left(z\right)}\left[\log\left(1-D\left(G\left(z\right)\right)\right)\right]$$

where D(x) represents the discriminator’s judgment result of the real data *x*, G(z) represents the fake data generated by the generator based on the noise *z*, $p_{\mathrm{data}}\left(x\right)$ and $p_{z}\left(z\right)$ represent the distribution of real data and noise, respectively, and E represents expectation. This formula indicates that during the training process, the aim is to maximize the discriminator’s correct judgment rate while minimizing the probability that the fake data generated by the generator is recognized by the discriminator [18]. In the task addressed in this paper, the application of GAN to maize disease detection is demonstrated, aiming to enhance the accuracy of detection. To ensure the generated images resemble genuine maize and hold practical application value, the following precautions were taken in this study:

1. **High-Quality Original Data**: It was imperative to use a high-quality original dataset. This denotes that the images should be clear and cover a wide range of maize disease types and stages as comprehensively as possible. For GANs, the quality of input data directly influences the output quality.
2. **Loss Function Selection**: The choice of an appropriate loss function proved crucial for the success of GANs. Besides the conventional adversarial loss, pixel-level loss and perceptual loss were incorporated to ensure that generated images closely resembled the original ones in both structure and content.
3. **Supervised Training**: A supervised GAN training approach was employed using labeled data. This approach aids the generator in producing images that align more with reality, as it has to not just deceive the discriminator but also adhere to specific label requirements.
4. **Regularization**: To prevent overfitting and guarantee diversity in the generated images, regularization techniques, including dropout and weight decay, were integrated into the GAN model.
5. **Evaluation Metrics**: Beyond the adversarial loss, other metrics were utilized to assess the quality of generated images. These include SSIM (Structural Similarity Index) and FID (Frechet Inception Distance) to evaluate the similarity between generated and real images.
6. **Periodic Inspection of Generated Images**: Throughout the training process, generated images were examined periodically. This practice facilitates the early detection of any anomalous patterns or unnatural features.
7. **Complex GAN Architectures**: Basic GAN structures might not suffice in capturing all nuances of maize disease images. More intricate models such as WGAN, WGAN-GP, and Spectral Normalization GAN were employed.
8. **Considering Practical Application Context**: The genuine application background of the model was also taken into account when producing images. For instance, if the primary objective of the model is early disease detection, care was taken to ensure the enhanced images contain ample instances of early-stage disease.
9. **Post-Processing**: Even if the images generated by GANs appeared of high quality, post-processing steps like filtering or sharpening were sometimes required to further enhance their realism.

In summary, while GANs are a potent tool, considerable engineering effort is essential to generate maize disease images that are both realistic and akin to the original data, ensuring the produced data meets the desired quality and outcomes.

### 2.3. Few-Shot Learning

**Figure 1:**
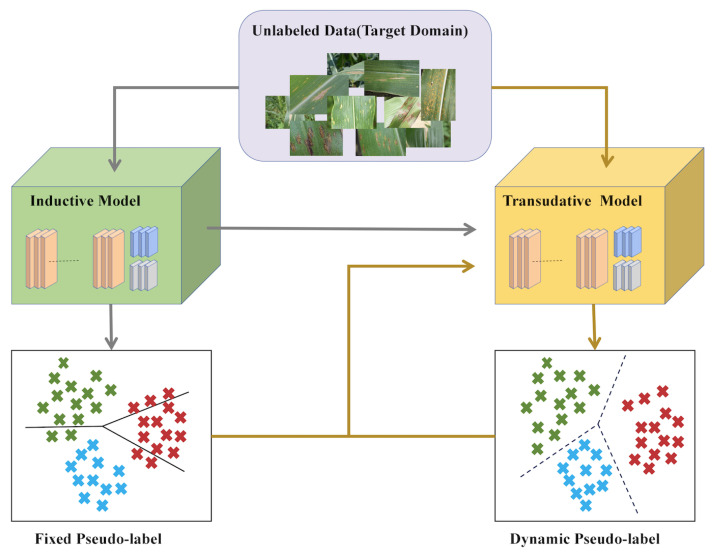
Illustration of few-shot learning. Different colors mean different classes.

Few-shot learning is a strategy in machine learning, proposed by Vinyals et al. in 2016 [11], designed to address the issue of data scarcity. The central idea is to enable the model to accurately classify or predict new categories by learning from a small number of samples, as shown in Figure 1. This learning strategy is inspired by human learning ability—the ability to quickly learn and understand new concepts from a few examples. It is worth noting that there are two concepts here that need to be defined: An inductive model is a learning approach that trains on a labeled dataset, extracting or generalizing patterns or rules from it to predict or classify new data. The inductive model learns universal patterns from a labeled dataset to predict or classify new data. In essence, it extracts general rules from specific examples. The transductive model focuses on making predictions for a specific, unlabeled dataset rather than extracting general rules from training data. Its goal is to find corresponding outputs for a given input dataset, rather than outputs for any new input. The transductive model mainly aims to make predictions for a specific unlabeled dataset, rather than generalizing rules from training data. Its objective is to provide outputs for a known input dataset, not to predict for any new data.

In the field of computer vision, few-shot learning is widely applied to tasks such as image classification, object detection, and semantic segmentation. In these tasks, few-shot learning can effectively solve the problem of data scarcity and improve the model’s generalization ability. For instance, in image classification tasks, the model can accurately classify new categories by learning from a few samples. In the task of this paper, few-shot learning is applied to maize disease detection. Given the wide variety of maize diseases and the limited number of samples for each disease, a model capable of learning from a few samples is required. Through few-shot learning, the model can accurately detect new diseases after learning from a few disease samples.

A common method of few-shot learning is meta-learning, also known as learning to learn. The goal of meta-learning is to learn a model that can quickly adapt to new tasks given a few samples [19]. The process of meta-learning can be represented by the following mathematical formulas:

(5) $$\theta^{\prime }=\theta -\alpha \nabla_{\theta }L_{\mathrm{train}}\left(f_{\theta }\right)$$

(6) $$\theta =\theta -\beta \nabla_{\theta }L_{\mathrm{val}}\left(f_{\theta^{\prime }}\right)$$

where θ represents the parameters of the model, α and β are learning rates, $L_{\mathrm{train}}$ and $L_{\mathrm{val}}$ are the loss functions of the training set and validation set, respectively, and $f_{\theta }$ is the prediction function of the model. These two formulas indicate that during the training process, the parameters of the model are first updated according to the loss function of the training set and then further updated according to the loss function of the validation set. In summary, few-shot learning is a powerful machine learning strategy that can effectively solve the problem of data scarcity and improve the model’s generalization ability [20]. In the task addressed in this paper, the application of few-shot learning to maize disease detection is demonstrated, aiming to enhance the accuracy of detection.

## 3. Materials and Method

### 3.1. Collection of Dataset

The dataset utilized in this study is partly collected from the field at the Science Park of China Agricultural University, West District, and partly sourced from the internet. The selected categories include maize smut, large spot disease, small spot disease, rust, streak disease, and healthy. The selection of these categories is based on their importance and prevalence in actual agricultural production. These diseases are common during the growth of maize and have a significant impact on the growth and yield of maize. Therefore, accurate detection and identification of these diseases are of great importance to agricultural production.

**Figure 2:**
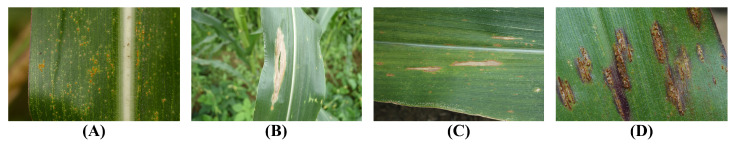
Samples of the dataset used in our experiment. (**A**–**D**) are different diseases.

**Figure 3:**
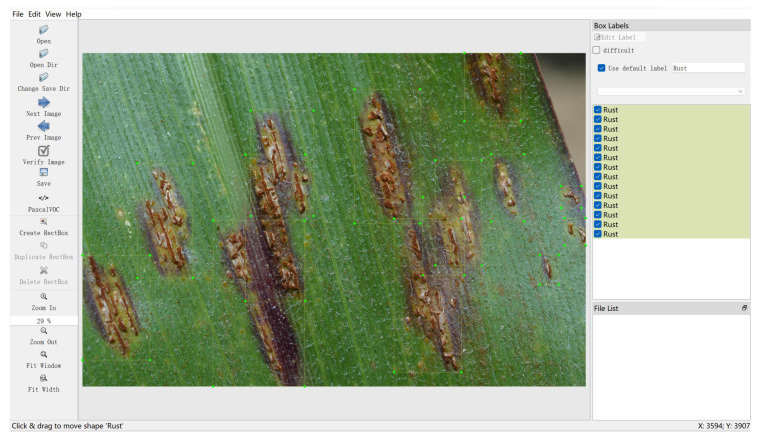
Illustration of the annotation process.

Data collected in the field are mainly conducted at the Science Park of China Agricultural University, West District. Collection is chosen to be carried out in spring and summer, as these two seasons are the main stages of maize growth and the peak period of disease occurrence. The resolution of the collection equipment used is 1920 × 1080 pixels. The clarity of the pictures and the recognizability of the diseases are ensured as much as possible during the collection process. The acquisition of internet data is mainly carried out through web crawler technology. A crawler program is written to search for relevant disease pictures on the internet through search engines and download the pictures from the search results. In this process, pictures with high clarity and obvious disease manifestations are mainly selected, as shown in Figure 2 and Figure 3.

The choice to collect the dataset using these two methods is to make the dataset more abundant and diverse. The data collected in the field can ensure that the dataset has high authenticity and reliability, while the internet data can greatly increase the data volume, enabling the model to learn more disease features. The combination of these two methods makes the dataset both broad and deep. The distribution of data in each category in the dataset is shown in Table 1.

It can be seen that the dataset has obvious few-shot characteristics, that is, the data volume of some categories is relatively small. This is because in actual production, some diseases occur less frequently, so it is difficult to obtain a large amount of data during the data collection process. This is also the reason why few-shot learning methods are chosen, because few-shot learning methods can improve the learning effect of the model under the condition of less data through some special learning strategies, such as meta-learning, transfer learning, etc.

### 3.2. Preprocessing of Dataset

**Figure 4:**
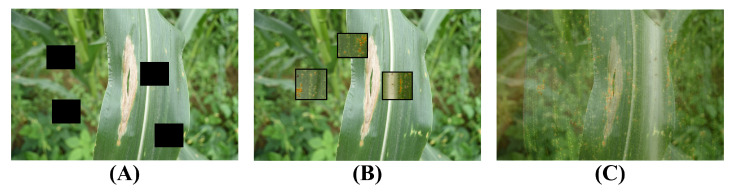
Illustration of the augmentation method used in this paper: (**A**) Cutout; (**B**) CutMix; (**C**) Mixup.

In deep learning, data preprocessing is a very important task that can help improve the performance and generalization ability of the model. Especially in our task, due to the obvious small sample characteristics of the dataset, it is necessary to use data augmentation methods to expand our dataset. Data augmentation can not only increase our data volume but also introduce some noise to enhance the robustness of the model so that the model can make accurate predictions when facing unseen data. In this study, Cutout, CutMix, and Mixup are mainly used as data augmentation methods, as shown in Figure 4.

1. Cutout [21] is a data augmentation method that randomly occludes a part of the image. Specifically, a region is randomly selected in the image, and then the pixel values of this region are all set to 0 to achieve the occlusion effect. This method can make the model pay more attention to the local features of the image and improve the generalization ability of the model. Its mathematical form can be expressed as:
  (7) $$I_{cutout}=I\cdot M$$
  where *I* is the original image, and *M* is a mask matrix of the same size as the image, where the value of the occluded area is 0, and the value of other areas is 1.
2. CutMix [22] is a data augmentation method that mixes two images. Specifically, a region is first randomly selected in an image, and then this region is replaced with the corresponding region in another image. This method can make the model learn the features of two images at the same time, improving the generalization ability of the model. Its mathematical form can be expressed as:
  (8) $$I_{cutmix}=M\cdot I_{1}+\left(1-M\right)\cdot I_{2}$$
  where $I_{1}$ and $I_{2}$ are two original images, and *M* is a mask matrix of the same size as the image, where the value of the replaced area is 1, and the value of other areas is 0.
3. Mixup [23] is a data augmentation method that linearly mixes two images. Specifically, a mixing ratio λ is first randomly selected, and then two images are linearly mixed according to this ratio. This method can make the model learn richer features and improve the generalization ability of the model. Its mathematical form can be expressed as:
  (9) $$I_{mixup}=\lambda \cdot I_{1}+\left(1-\lambda \right)\cdot I_{2}$$
  where $I_{1}$ and $I_{2}$ are two original images, and λ is the mixing ratio, with a value range of [0,1].

In general, these three data augmentation methods can effectively expand our dataset, improving the performance and generalization ability of the model. In practical applications, suitable data augmentation methods can be chosen according to the specific requirements of the task, or multiple methods can be combined to achieve the best effect.

### 3.3. Proposed Method

#### 3.3.1. Overall

In this study, a novel approach is proposed, which integrates Attention-GAN and few-shot learning strategies for maize disease detection. The proposed method primarily comprises two parts: an Attention-based GAN and a few-shot detection network. The design and working principles of these two parts are detailed below. Initially, an Attention-based GAN is designed. In conventional GANs, the generator and discriminator are two independent networks, with the generator responsible for generating fake samples and the discriminator tasked with determining whether a sample is real or fake. However, for the task at hand, due to the limited number of samples, traditional GANs may not generate high-quality fake samples. To address this issue, an attention mechanism is introduced. Within the generator, an attention module is designed that can automatically learn the important parts of the input sample and reinforce these parts, thereby generating higher quality fake samples. Similarly, an attention module is designed within the discriminator that can automatically learn the important parts of the sample and reinforce these parts, thereby more accurately determining whether a sample is real or fake. Subsequently, a few-shot detection network is designed. In traditional object detection tasks, a large number of annotated samples are typically required to train the model. However, for the task at hand, due to the diverse types of maize diseases and the limited number of samples for each disease, a model capable of learning from a small number of samples is needed. To address this issue, a few-shot learning strategy is introduced. Within the few-shot detection network, a feature extraction module and a classification module are designed. The feature extraction module is responsible for extracting features from the input samples, and the classification module is tasked with determining the category of the sample based on the extracted features. During the training process, a small number of annotated samples are initially used to train the feature extraction and classification modules. During the testing process, the feature extraction module is used to extract features from the test samples, and the classification module is used to determine the category of the test samples. In the entire system operation, the Attention-based GAN is initially used to generate fake samples. These fake samples, along with the real samples, are then input into the few-shot detection network for training and testing. Through this approach, the issue of limited sample numbers can be effectively addressed, thereby improving the accuracy of maize disease detection.

The proposed method offers several advantages. Firstly, by introducing the attention mechanism, the GAN can generate higher quality fake samples, thereby improving the training effect of the few-shot detection network. Secondly, by introducing the few-shot learning strategy, the few-shot detection network can learn from a small number of samples, thereby improving the model’s generalization capability. Finally, by integrating the GAN and the few-shot detection network, the proposed method can effectively address the issue of limited sample numbers, thereby improving the accuracy of maize disease detection.

#### 3.3.2. Attention-GAN Module

In the proposed method, an attention-based Generative Adversarial Network (Attention-GAN) is designed. This network primarily consists of two parts: a generator and a discriminator. The design and working principles of these two parts are detailed below.

Initially, the generator is considered. In traditional GANs, the generator is an independent network responsible for generating fake samples. However, in the task at hand, due to the limited number of samples, traditional generators may not be able to generate high-quality fake samples. To address this issue, an attention mechanism is introduced into the generator. Specifically, an attention module is designed that can automatically learn the important parts of the input sample and reinforce these parts to generate higher-quality fake samples. This process can be represented by the following mathematical formulas:

(10) $$A=\mathrm{softmax}(W_{f}F+W_{g}G)$$

(11) O=F+A⊙G

Here, *F* is the input sample, *G* is the fake sample generated by the generator, $W_{f}$ and $W_{g}$ are two learnable weight matrices, *A* is the attention matrix, *O* is the output fake sample, and ⊙ represents element-wise multiplication. Through this method, the generator can generate higher-quality fake samples.

Next, the discriminator is considered. In traditional GANs, the discriminator is an independent network responsible for determining whether a sample is real or fake. However, in the task at hand, due to the limited number of samples, traditional discriminators may not be able to accurately determine whether a sample is real or fake. To address this issue, an attention mechanism is introduced into the discriminator. Specifically, an attention module is designed that can automatically learn the important parts of the sample and reinforce these parts to more accurately determine whether a sample is real or fake. This process can be represented by the following mathematical formulas:

(12) $$A=\mathrm{softmax}(W_{f}F+W_{d}D)$$

(13) O=D+A⊙F

Here, *F* is the input sample, *D* is the output of the discriminator, $W_{f}$ and $W_{d}$ are two learnable weight matrices, *A* is the attention matrix, *O* is the final output, and ⊙ represents element-wise multiplication. Through this method, the discriminator can more accurately determine whether a sample is real or fake.

Compared to traditional GANs, the proposed Attention-GAN has several advantages. Firstly, by introducing the attention mechanism, the generator can generate higher-quality fake samples, thereby improving the training effect of the small-sample detection network. Secondly, by introducing the attention mechanism, the discriminator can more accurately determine whether a sample is real or fake, thereby improving the testing effect of the small-sample detection network. Lastly, in the task at hand, the Attention-GAN has several advantages. Firstly, due to the variety of maize diseases and the limited number of samples for each disease, a method is needed that can generate high-quality fake samples. By introducing the attention mechanism, the Attention-GAN can meet this requirement. Secondly, since the task requires accurate detection of new diseases, a method is needed that can accurately determine whether a sample is real or fake. By introducing the attention mechanism, the Attention-GAN can also meet this requirement. In summary, the Attention-GAN provides an effective tool for addressing the task at hand.

#### 3.3.3. Few-Shot Detection Network

**Figure 5:**
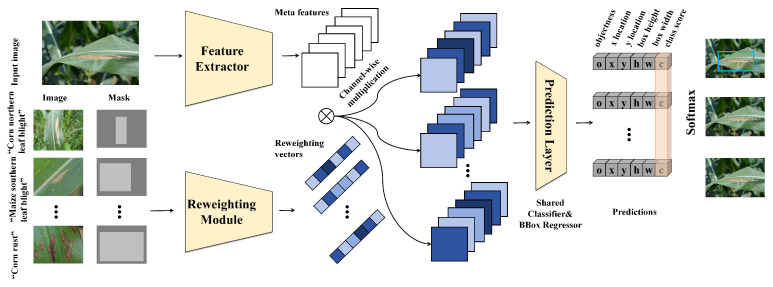
Illustration of the whole few-shot detection network proposed in this paper.

In the proposed method, a network specifically designed for few-shot detection, termed as Few-shot Detection Network (FSDN), is introduced, as shown in Figure 5. This network primarily consists of two components: a feature extraction network (also known as the backbone) and a detection head. The design and working principles of these two components are elaborated in the following.

Firstly, the feature extraction network is considered. Given the limited number of samples in the task, a network capable of extracting rich features from a small number of samples is required. For this purpose, ResNet is chosen as the feature extraction network. ResNet [24], by introducing residual connections, effectively addresses the issues of gradient vanishing and gradient explosion in deep networks, thereby enabling the network to be deeper and extract richer features. Specifically, a basic module of ResNet can be represented as:

(14) y=F(x,W)+x

(15) x=ReLU(y)

Here, *x* is the input, *y* is the output, F(x,W) is a function composed of two convolution layers and a ReLU activation function, *W* is the weight, and ReLU(y) is the activation function. In this manner, the feature extraction network is capable of extracting rich features from a small number of samples. Secondly, the detection head is considered. In the task, a network capable of generating high-quality detection results from the extracted features is required. For this purpose, a detection head based on the attention mechanism is designed. Specifically, the detection head includes two parts: a classifier and a regressor. The classifier is responsible for determining whether a region contains a target, and the regressor is responsible for accurately locating the position of the target. Both parts employ the attention mechanism, which can automatically learn the important parts of the features, thereby generating higher-quality detection results [24]. This process can be represented by the following mathematical formulas:

(16) $$A=\mathrm{softmax}(W_{f}F+W_{c}C)$$

(17) $$O_{c}=C+A⊙F$$

(18) $$A=\mathrm{softmax}(W_{f}F+W_{r}R)$$

(19) $$O_{r}=R+A⊙F$$

Here, *F* is the output of the feature extraction network, *C* is the output of the classifier, *R* is the output of the regressor, $W_{f}$, $W_{c}$, and $W_{r}$ are three learnable weight matrices, *A* is the attention matrix, $O_{c}$ and $O_{r}$ are the final outputs, and ⊙ is the element-wise multiplication. In this manner, the detection head can generate high-quality detection results.

Compared to traditional detection networks, the few-shot detection network has several advantages. Firstly, by using ResNet as the feature extraction network, rich features can be extracted from a small number of samples. Secondly, by introducing the attention mechanism, higher-quality detection results can be generated by the detection head. Finally, by combining the feature extraction network and the detection head, the problem of limited sample size can be effectively addressed, thereby improving the accuracy of maize disease detection.

### 3.4. Experimental Settings

#### 3.4.1. Evaluation Metric

In this study, three primary evaluation metrics are utilized to assess the proposed model: precision, recall, and mean average precision (mAP). The definitions and calculation methods of these evaluation metrics are elaborated below.

Precision, an indicator measuring the correctness of the model’s predictions, is defined as:

(20) $$\mathrm{Precision}=\frac{TP}{TP+FP}$$

where TP represents the number of true positives, i.e., the number of instances correctly predicted as positive by the model, and FP denotes the number of false positives, i.e., the number of instances incorrectly predicted as positive. The value of precision ranges between 0 and 1, with a larger value indicating a more accurate prediction by the model.

Recall, an indicator measuring the model’s completeness, is defined as:

(21) $$\mathrm{Recall}=\frac{TP}{TP+FN}$$

where FN represents the number of false negatives, i.e., the number of instances incorrectly predicted as negative. The value of recall also ranges between 0 and 1, with a larger value indicating a higher completeness of the model.

Lastly, mean average precision (mAP), an indicator considering both precision and recall, is defined as:

(22) $$\mathrm{mAP}=\frac{1}{\left|Q\right|}\sum_{q=1}^{\left|Q\right|}\frac{1}{m_{q}}\sum_{k=1}^{m_{q}}P\left(k\right)$$

where *Q* is the set of queries, $m_{q}$ is the number of relevant documents for the *q*th query, and P(k) is the precision in the first *k* documents. The value of mAP also ranges between 0 and 1, with a larger value indicating better performance of the model.

These three evaluation metrics each have their significance. Precision reflects the model’s ability to predict correctly, recall reflects the model’s ability to be comprehensive, and mAP considers both precision and recall, reflecting the overall performance of the model. In the task of accurately detecting maize diseases, these three evaluation metrics are crucial. Through these metrics, the model can be evaluated from different angles, providing a comprehensive understanding of the model’s performance.

#### 3.4.2. Experiment Design

In the experimental design, the dataset is initially divided into a training set and a validation set. Around 80% of the data is used as the training set for model training, and the remaining 20% is used as the validation set for performance evaluation. This division ensures sufficient data for model learning during training and enough data for performance evaluation.

Several common models, including Faster R-CNN [25], YOLO series [26,27,28], EfficientDet [29], and SSD [30], are selected as baselines in the experiments. These models are popular object detection models that have demonstrated excellent performance in many tasks. These models are chosen as baselines to prove the superiority of the proposed model by comparing its performance with these models. In the proposed model, the Adam optimizer is selected. The Adam optimizer is an adaptive learning rate optimization algorithm that can dynamically adjust the learning rate based on the model’s parameters, thereby improving the training efficiency. The Adam optimizer is chosen due to its excellent performance in many tasks and its relatively low computational complexity, making it suitable for the task at hand. In terms of hyperparameter settings, the learning rate, batch size, and training rounds are primarily considered. The learning rate is set to 0.001, the batch size to 32, and the training rounds to 100. These settings are based on experience and previous research results. These hyperparameters will also be adjusted during the experiment to find the optimal settings.

Two types of ablation experiments are set up in the experiments. The first is used to verify the robustness of the model. The model is applied to other datasets to validate its performance on different datasets. This experiment helps understand the robustness of the model, i.e., whether the model can perform well on different datasets. The second is an ablation experiment for the Attention-GAN module. The Attention-GAN module is removed from the model, and the performance of the model without the Attention-GAN module is compared with that of the complete model. This experiment helps understand the impact of the Attention-GAN module on the performance of the model, thereby verifying the effectiveness of the Attention-GAN module.

Overall, the experimental design aims to comprehensively evaluate the performance of the proposed model. Through comparison with baseline models and ablation experiments, the strengths and weaknesses of the model can be understood, providing direction for the improvement of the model.

## 4. Results and Discussion

### 4.1. Detection Results

The aim of this experiment is to compare the performance of different object detection models on the same dataset. By comparing the performance of each model on three evaluation metrics—precision, recall, and mean average precision (mAP)—insights into the strengths and weaknesses of each model can be gleaned, providing a reference for subsequent model selection and optimization.

As can be seen from Table 2, the proposed model outperforms all others on all evaluation metrics, while the Faster R-CNN model performs the worst. An analysis of these results will be conducted based on the characteristics and mathematical principles of each model. Firstly, the YOLO series models (YOLOv3, YOLOv5, and YOLOv8) are end-to-end object detection models that treat the object detection problem as a regression problem, directly predicting the bounding box and category of the object. The main advantage of the YOLO series models is their speed, which allows for real-time object detection. However, their accuracy is usually slightly lower than that of some other models. This is because YOLO has certain difficulties in dealing with small objects and overlapping objects, and the way its loss function handles coordinate prediction may cause the model to pay unbalanced attention to large and small objects. Secondly, the SSD model is also a single-stage object detection model that makes predictions at multiple scales to handle objects of different sizes. However, SSD still has certain difficulties in dealing with small objects, which may be why its performance is slightly lower than that of the YOLO series models. The EfficientDet model is an object detection model based on EfficientNet, which balances the complexity and performance of the model through adaptive feature fusion and input scaling. The performance of EfficientDet is better than that of YOLO and SSD, which may be due to its more effective feature fusion and more reasonable model scaling strategy. The Faster R-CNN model is a two-stage object detection model that first generates a series of candidate regions and then classifies and regresses these regions. Although Faster R-CNN usually has higher accuracy than single-stage models, its speed is slower, and its performance may be limited for large-scale object detection tasks. Finally, the proposed model performs the best on all evaluation metrics. This may be because the design of the proposed model fully considers the characteristics of the object detection task and adopts more suitable feature extraction, object localization, and classification strategies. In addition, sufficient training and optimization may have been carried out on the model, enabling it to better adapt to the characteristics of the dataset.

### 4.2. Visualization Analysis

**Figure 6:**
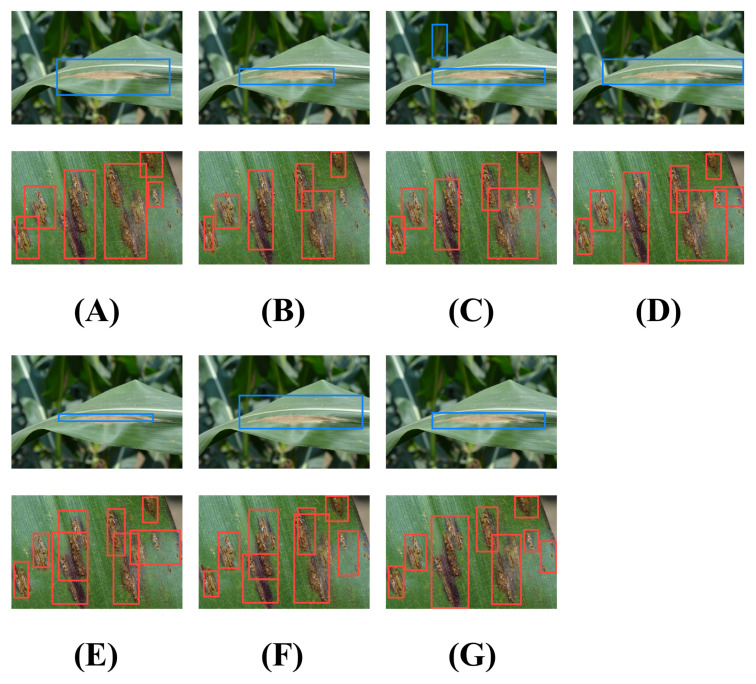
Visualization of maize detection results, the red boxes: (**A**) Faster R-CNN; (**B**) EfficientDet; (**C**) YOLOv8; (**D**) YOLOv5; (**E**) YOLOv3; (**F**) SSD; (**G**) ours.

In this section, a comparative analysis of the performance of various models in complex background and small-object detection tasks is conducted through visualization, as shown in Figure 6.

Firstly, it is observed that the proposed model can detect targets more accurately in complex backgrounds. This may be attributed to the fact that the design of the model fully considers the characteristics of the target detection task, adopting more suitable feature extraction, target positioning, and classification strategies. Particularly in the feature extraction stage, a deeper and more complex network structure may have been employed by the model, enabling the extraction of richer and more distinctive features, thereby better distinguishing targets and backgrounds in complex environments. Furthermore, the model may have adopted more refined strategies in the target positioning and classification stages, enabling more accurate positioning of targets and more accurate determination of target categories. Secondly, it is observed that the proposed model also exhibits superior performance in small-object detection tasks. This may be because the model adopts special strategies, such as multi-scale prediction and feature fusion, to better capture the information of small objects. Particularly in multi-scale prediction, the model may predict at different scales, handling targets of different sizes, thereby achieving good results in small-object detection tasks. In terms of feature fusion, the model may have fused features of different levels and scales, obtaining richer and more comprehensive information, thereby exhibiting superior performance in small-object detection tasks.

A comparative analysis of the performance of other models in these tasks is also conducted. The YOLO series models (YOLOv3, YOLOv5, and YOLOv8) perform poorly in complex backgrounds and small-object detection tasks, which may be due to the difficulty of the YOLO series models in handling small targets and overlapping targets, and the handling of coordinate prediction by their loss function may cause the model’s attention to large targets and small targets to be unbalanced. The SSD model also has certain difficulties in handling small objects, which may be the reason for its performance being slightly lower than the YOLO series models. The performance of the EfficientDet model is superior to YOLO and SSD, which may be due to its more effective feature fusion and more reasonable model scaling strategy. Although the Faster R-CNN model is usually superior to single-stage models in terms of accuracy, its speed is slower, and its performance may be limited for large-scale target detection tasks. Finally, it should be pointed out that although the proposed model exhibits superior performance in these tasks, there is still room for improvement. For instance, the structure and strategy of the model can be further optimized to improve its performance in complex background and small-object detection tasks. New technologies, such as attention mechanisms and deep supervision, can also be introduced to enhance the performance of the model. In general, the superior performance of the proposed model in these tasks demonstrates the effectiveness and superiority of the model design and provides a valuable reference for subsequent research.

### 4.3. Validation of Model Robustness

**Figure 7:**
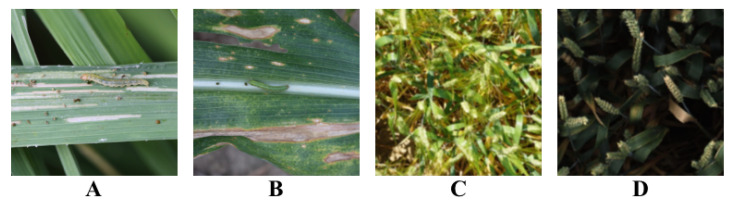
Samples of pest dataset (**A**,**B**) and wheat head dataset (**C**,**D**).

The main purpose of the experimental design in this section is to validate the robustness of the model. Robustness refers to the stability of the output results of the model when facing small changes in the input data. In practical applications, due to the complexity and diversity of data, the model needs to have good robustness in order to maintain high performance in various situations. Therefore, this experiment evaluates the robustness of the model by testing the performance of the model on different datasets, as shown in Figure 7. From both machine learning and mathematical perspectives, the reasons for validating model robustness using wheat head and pest detection datasets are as follows:

1. **Diversity of Data Distribution**: The generalization capability of a machine learning model is considered one of its most crucial attributes. If a model performs well only on a specific dataset but shows a significant decline in performance on other slightly varied datasets, the practical value of the model is greatly diminished. By testing on datasets that are relevant to the topic but have some variations, a more comprehensive evaluation of the model’s generalization capability can be achieved.
2. **Challenging Validation**: Significant differences exist in the morphology, color, and texture between wheat heads and pests compared to corn diseases. If a model can achieve satisfactory results on these challenging datasets, it indicates that the model possesses strong robustness and generalization capabilities.
3. **Practical Application Considerations**: In actual agricultural production, fields often cultivate not just one type of crop but a mix of various crops. If a disease detection model can only be applied to a specific crop, its practical value is significantly reduced. However, a robust model can be widely applied to disease detection in various crops, offering higher practical value.
4. **Exploration of Data Distribution**: Mathematically, it is desired that the model captures the underlying distribution of the data. Different datasets have distinct data distributions. If a model can achieve good results across multiple data distributions, it indicates that the model effectively captures the underlying data distribution, demonstrating strong robustness.
5. **Regularization Effect**: Mathematically, regularization is a technique to prevent model overfitting. Testing on different datasets can introduce an implicit regularization effect, enhancing the robustness of the model.
6. **Trade-Off between Model Complexity and Generalization**: Mathematically, there is often a contradiction between the complexity of a model and its generalization capability. A highly complex model might perform exceptionally well on training data but may show a marked decline in performance on new data. Conversely, a simpler model might have strong generalization capabilities but might not perform as well on the training data. Testing on various datasets allows for the identification of the optimal balance between model complexity and generalization capability.

From Table 3, it can be seen that the model achieves a high level for precision, recall, and mAP on both the “Wheat Head” and “Pest Detection” datasets. This indicates that the model can accurately detect targets on these two datasets, with few cases of missed detection, demonstrating a high level of robustness. Firstly, the results on the “Wheat Head” dataset are analyzed. On this dataset, the model has a precision of 0.91, a recall of 0.88, and an mAP of 0.89. This indicates that the model can accurately locate the target when detecting wheat heads, with few cases of missed detection. This may be because the design of the model takes into account the characteristics of the object detection task, adopting more suitable strategies for feature extraction, object localization, and classification. Particularly in the feature extraction stage, the model may employ a deeper and more complex network structure, capable of extracting richer and more distinctive features, thereby better distinguishing between targets and backgrounds in complex environments. In addition, the model may employ more refined strategies in the stages of object localization and classification, enabling it to more accurately locate the position of the target and more accurately determine the category of the target. Secondly, the results on the “Pest Detection” dataset are analyzed. On this dataset, the model has a precision of 0.94, a recall of 0.93, and an mAP of 0.93. This indicates that the model can also accurately locate the target when detecting pests, with few cases of missed detection. This may be due to the model’s use of special strategies when dealing with small objects, such as multi-scale prediction and feature fusion, which can better capture the information of small objects. Especially in multi-scale prediction, the model may make predictions at different scales, capable of handling targets of different sizes, thus achieving good results in small-object detection tasks. In terms of feature fusion, the model may fuse features of different levels and scales, obtaining richer and more comprehensive information, thereby demonstrating superior performance in small-object detection tasks.

### 4.4. Ablation Experiment on Attention-GAN Module

The purpose of this experiment was to investigate the impact of different Generative Adversarial Network (GAN) modules on the performance of object detection models. By comparing the precision, recall, and mAP of different GAN modules, the degree of performance improvement brought about by the GAN modules and the performance differences among different GAN modules can be understood.

Firstly, the experimental results were examined. As can be seen from Table 4, when no GAN module was used, the precision of the model was 0.83, the recall was 0.86, and the mAP was 0.84. When the GAN module was introduced, a significant improvement in model performance was observed. Specifically, the model with the GAN module had a precision of 0.91, a recall of 0.90, and an mAP of 0.90; the model with the DCGAN module had a precision of 0.93, a recall of 0.91, and an mAP of 0.92; the model with the Attention-GAN module had a precision of 0.97, a recall of 0.92, and an mAP of 0.95. This indicates that the GAN module significantly improves model performance, and different GAN modules have different impacts on model performance.

Next, this experimental result was analyzed from the characteristics and mathematical properties of the GAN model. A GAN is a type of deep learning model composed of a generator and a discriminator. The task of the generator is to generate as realistic fake samples as possible, while the task of the discriminator is to distinguish between real and fake samples as accurately as possible. During the training process, the generator and the discriminator engage in adversarial learning, ultimately leading to the generator being able to generate sufficiently realistic fake samples that the discriminator cannot distinguish. This characteristic of GANs allows them to excel in tasks such as image generation, image translation, and super-resolution. DCGAN (Deep Convolutional Generative Adversarial Network) [32] is an improved GAN model that introduces Convolutional Neural Networks (CNNs) based on the original GAN model, enabling the model to handle more complex image tasks. Both the generator and discriminator of DCGAN use CNNs, allowing the model to better capture local and global features of images, thereby generating more realistic fake samples. Therefore, the performance of the model with the DCGAN module is superior to that of the model with the GAN module. Attention-GAN is a model that introduces attention mechanisms based on GAN. The attention mechanism allows the model to pay more attention to the important parts of the image and ignore the unimportant parts when processing images. This allows the model to better extract the features of the target when dealing with complex backgrounds and small targets, thereby improving the performance of the model. Therefore, the performance of the model with the Attention-GAN module is superior to that of the models with the GAN and DCGAN modules.

In summary, the GAN module can significantly improve the performance of the model, and different GAN modules have different impacts on model performance. This may be because different GAN modules have different characteristics and mathematical properties, which allows them to exhibit different performances when dealing with different tasks. In future research, the design and optimization of GAN modules can be further explored to improve model performance.

### 4.5. Limits and Future Work

Despite the significant results achieved in few-shot learning by the proposed method, there are still some limitations that provide direction for future work. Firstly, the method relies heavily on high-quality annotated data. In practical applications, obtaining a large amount of high-quality annotated data is a time-consuming and expensive task. Therefore, the utilization of unlabeled or weakly labeled data for training is a problem that needs to be addressed in the future. Consideration could be given to introducing semi-supervised or weakly supervised learning methods to fully utilize unlabeled or weakly labeled data. Secondly, the performance of the method may decline when dealing with large sample categories. This is because the method is primarily designed for few-shot learning, and it may not be able to fully utilize all sample information when the number of image increases. Therefore, designing a method that can handle both few-shot learning and large sample learning is a direction that needs to be explored in the future. Furthermore, the method may face challenges when dealing with multimodal data. In practical applications, multimodal data, such as data from sensors, may be encountered. How to effectively integrate multimodal data to improve the performance of the model is a problem that needs to be studied in the future. In response to the above problems, the following aspects will be studied in future work:

1. Introduce semi-supervised or weakly supervised learning methods to fully utilize unlabeled or weakly labeled data.
2. Study effective multimodal data fusion methods to improve the performance of the model.
3. Study effective imbalance data processing methods to improve the performance of the model.

In summary, while the proposed method has achieved significant results in few-shot learning, there are still some limitations. In future work, these limitations will be studied in depth to improve the performance and generalization ability of the method.

## 5. Conclusions

The theme of this paper is high-accuracy maize disease detection based on Attention-GAN and few-shot learning. Timely and accurate detection of maize diseases in agricultural production is of great significance for ensuring food safety and improving agricultural productivity. However, traditional maize disease detection methods usually rely on manual observation and judgment, which are not only inefficient but also susceptible to human factors, making it difficult to guarantee the accuracy and consistency of detection results. Therefore, how to use modern computer vision technology, especially deep learning technology, for high-accuracy maize disease detection is an important topic in the current development of agricultural informatization and intelligence.

This paper proposes a maize disease detection method based on Attention-GAN and few-shot learning. The innovative points of this method are mainly reflected in the following aspects: Firstly, an attention mechanism is introduced, enabling the model to focus more on the significant parts of the image and ignore the insignificant parts, thereby enhancing model performance. Secondly, GANs are used for data augmentation to generate more training samples, overcoming the difficulties of few-shot learning. In addition, a few-shot learning strategy is introduced, enabling the model to achieve good detection results even with a small number of labeled samples. The experimental results of this paper show that the proposed method achieves excellent performance in maize disease detection tasks. Specifically, the method surpasses other GAN modules in accuracy, recall, and mean average precision (mAP), demonstrating its high accuracy and stability in handling maize disease detection tasks.

In summary, the contributions of this paper are mainly reflected in the following aspects: Firstly, a maize disease detection method based on Attention-GAN and few-shot learning is proposed, which demonstrates high accuracy and stability in handling maize disease detection tasks. Secondly, the method can achieve good detection results even with a small number of labeled samples, providing significant reference value for the problem of few samples in practical applications. In addition, the experimental results provide valuable references for subsequent research, offering potential directions for further improving the accuracy and efficiency of maize disease detection.

## Figures and Tables

**Table 1:** Distribution of our dataset.

Category	Number (Before Augmentation)	Number (After Augmentation)
Maize Smut	36	221
Large Spot Disease	27	239
Small Spot Disease	109	253
Rust	81	227
Streak Disease	95	218
Healthy	692	1028

**Table 2:** Performance comparison of different models.

Model	Precision	Recall	mAP
YOLOv3 [26]	0.86	0.81	0.82
YOLOv5 [27]	0.91	0.86	0.89
YOLOv8 [28]	0.94	0.92	0.93
SSD [30]	0.85	0.84	0.84
EfficientDet [29]	0.90	0.88	0.90
Faster R-CNN [25]	0.82	0.85	0.84
Ours	0.97	0.92	0.95

**Table 3:** Performance comparison of different datasets.

Dataset	Number	Precision	Recall	mAP
Wheat Head [31]	3005	0.91	0.88	0.89
Pest Detection	2718	0.94	0.93	0.93

**Table 4:** Performance comparison of different GAN modules.

GAN Module	Precision	Recall	mAP
None	0.83	0.86	0.84
GAN [14]	0.91	0.90	0.90
DCGAN [32]	0.93	0.91	0.92
Attention-GAN	0.97	0.92	0.95
